# Hematologic Rescue of CAR T-cell–mediated Prolonged Pancytopenia Using Autologous Peripheral Blood Hematopoietic Stem Cells in a Lymphoma Patient

**DOI:** 10.1097/HS9.0000000000000545

**Published:** 2021-02-17

**Authors:** Philipp Gödel, Noëlle Sieg, Jan-Michel Heger, Nadine Kutsch, Carmen Herling, Ben-Niklas Bärmann, Christof Scheid, Peter Borchmann, Udo Holtick

**Affiliations:** 1 Cologne Lymphoma Working Group, Department of Internal Medicine, Medical Faculty and University Hospital of Cologne, University of Cologne, Cologne, Germany; 2 Center for Integrated Oncology Aachen Bonn Köln Düsseldorf (CIO ABCD), Germany; 3 Klinik für Hämatologie, Onkologie und klinische Immunologie, Universitätsklinikum Düsseldorf, Düsseldorf, Germany.

We report on a case of severe prolonged pancytopenia after *CD19* CAR T-cell (CART) therapy for aggressive lymphoma that successfully resolved after autologous peripheral blood stem cell transplantation (ASCT).

A 51-year-old female suffered from germinal center-type double-hit diffuse large B-cell lymphoma (DLBCL). Besides hypertension, deep vein thrombosis, and hypothyroidism, she had no relevant preexisting conditions. Beginning in December 2018, she received 6 cycles of rituximab, cyclophosphamide, doxorubicin, vincristine, prednisolone chemotherapy and obtained complete remission but relapsed only 4 months later. Salvage chemotherapy with platinum-containing regimens (2x rituximab, dexamethason, high-dose ara-C, cisplatin, 1x rituximab, gemcitabine, dexamethason, cisplatin [R-GDP]) achieved a partial remission that was consolidated by high-dose chemotherapy (rituximab, thiotepa, ara-C, etoposide) followed by ASCT. However, she relapsed again only 3 months later. After insufficient response to 1 cycle each of R-GDP and R-bendamustine/polatuzumab-vedotin, she obtained partial remission after 1 cycle of rituximab, ifosfamide, carboplatin, etoposide with significant persisting tumor masses (periuterine lesion of 4.9 × 4.6 cm) on computed tomography (CT) scans.

At the end of July 2019, she was in good general condition (Eastern Cooperative Oncology Group 0) with treatment-related anemia and thrombocytopenia but normal leukocyte counts and received *CD19*-targeting CART treatment (tisagenlecleucel) after lymphodepleting chemotherapy with fludarabine/cyclophosphamide (30 and 300 mg/m^2^, respectively, q3d).

One day before CART infusion, she developed neutropenic fever without evident infection and sterile blood cultures. Antibiotic therapy with piperacillin/tazobactam was discontinued after 4 days when the patient staid afebrile and C-reactive protein (CRP) remained at low levels.

A second febrile episode (up to 41.2°C) beginning at day 4 was followed by a sharp increase in inflammatory parameters (interleukin 6 [IL-6] max. 4071 ng/L, norm < 8 ng/L) and strong CART expansion as measured per quantitative polymerase chain reaction (PCR) (max. 5.6 × 10^6^ copies per million white blood cells [WBC]), again without evidence of infection and negative blood cultures. Meropenem was administered due to ongoing severe neutropenia and extended spectrum beta-lactamase colonization in rectal swabs. She was transferred to our intermediate care unit for monitoring of cytokine release syndrome (CRS) grade III (American Society of Blood and Marrow Transplantation criteria) without signs of neurotoxicity. Two doses of tocilizumab 400 mg and low-dose norepinephrine were given at day 6. Thereafter, she became afebrile and was transferred back to our hematologic ward at day 10.

However, severe pancytopenia remained. At day 13, daily administration of lenograstim 33.6 Mio IE s.c. was commenced, but no sustained bone marrow recovery was noted. Bone marrow aspirate at day 31 showed a considerably hypocellular marrow with few mature lymphocytes and no evidence of lymphomatous infiltration on cytology review (Figure 1). Inflammatory markers such as IL-6, ferritin, and CRP had returned to normal or low levels; thus, inflammation was not considered to be involved in the ongoing pancytopenia.

**Figure 1.**
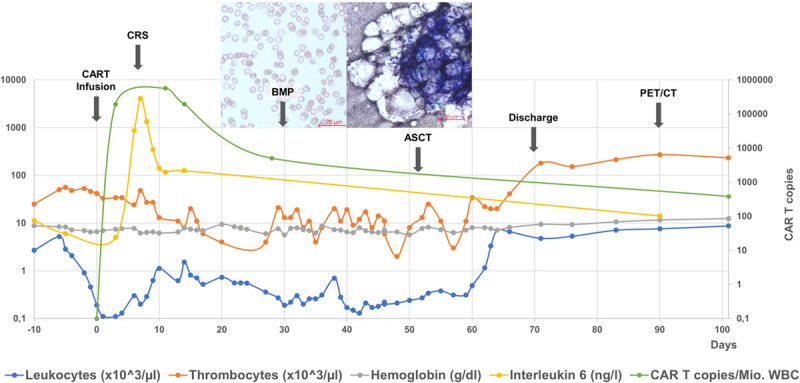
**Blood counts (leucocytes/thrombocytes × 103/μL, hemoglobin g/dl), serum IL-6 (ng/l), CART expansion (copies per million WBC), bone marrow cytology, and major clinical events (arrows).** ASCT = autologous peripheral blood stem cell transplantation; BMP = bone marry puncture; CAR = chimeric antigen receptor; CART = CAR T cell; CRS = cytokine release syndrome; PET/CT = positron emission tomography/computed tomography; WBC = white blood cell.

Folic acid and vitamin B12 levels were within normal range and PCR of peripheral blood for Ebstein-Barr virus, cytomegalovirus, Herpes simplex virus, varizella zoster virus, and Parvovirus B19 remained negative. Prophylactic cotrimoxazole and valaciclovir were discontinued without any effect on bone marrow recovery.

A positron emission tomography (PET)/CT scan at day 28 showed a good partial metabolic remission without evidence of bone marrow involvement of DLBCL. In the further course, the patient suffered from catheter-associated thrombosis and *Escherichia coli* sepsis that was again treated with meropenem.

At day 52, in light of ongoing severe pancytopenia (leukocytes 0.27 × 10^9^/L, Hb 7.7 g/dL, platelets 13 × 10^9^/L), we infused 4.63 × 10^6^/kg body weight CD34^+^ cryopreserved autologous peripheral blood hematopoietic stem cells left from the first stem cell collection. Twelve days later, under continued granulocyte colony-stimulating factor stimulation, the patient’s leukocyte counts had recovered to normal without need of further thrombocyte transfusions, 68 days after initiation of lymphodepleting chemotherapy. PET/CT staging 3 months after CART therapy revealed ongoing partial remission of the abdominal bulk but 1 new infrarenal PET positive lesion that showed small areas of proliferating CD19^+^ lymphoma cells among mostly necrotic lymphoid tissue on histology review. CARTs remained detectable at a low level (5.0 × 10^3^ copies per million WBC). Although irradiation was planned, further progression involving the peritoneum was noted, relapsed DLBCL did not respond to rituximab/lenalidomide, and the patient was switched to rituximab, ifosphamide, gemcitabine, vinorelbine, which she is now responding to. Consolidation by allogeneic stem cell transplantation is scheduled.

To the best of our knowledge, this is the first report of a DLBCL patient receiving ASCT as salvage after prolonged cytopenia following CART infusion. The only published report so far discusses the case of a 56-year-old multiple myeloma patient who was treated with anti-B-cell maturation antigen CART therapy. He suffered from Common Terminology Criteria for Adverse Events grade 4 cytopenia for 45 days accompanied by CRS grade 2. Ten days after, ASCT blood counts recovered and the patient achieved very good partial remission at day 105.^1^

Prolonged cytopenia is a common finding in *CD19*-targeting CART therapy^2,3^ with up to 29% and 42% of 31 lymphoma patients experiencing grade 3-4 neutropenia and thrombocytopenia after axicabtagene ciloleucel,^4^ which is consistent with data from the pivotal ZUMA-1 (17% ≥3 cytopenias at 3 mo)^5^ and JULIET (24% ≥3 cytopenias at 28 d)^6^ trials. In our own cohort of patients treated with commercially available *CD19*-directed chimeric antigen receptor (CAR) T-cells, 9/35 patients (26%) experienced prolonged neutropenia. Median duration of grades 3 and 4 neutropenia in these patients was 33 days (15–66 d) and 11 days (2–47 d, unpublished results).

The underlying mechanisms remain poorly understood. Higher grade CRS with or without neurotoxicity^2,4,7^ consistently correlated with prolonged cytopenias. In a cohort of 41 patients suffering from lymphoma, leukemia, and myeloma, correlation of CRS and high inflammatory parameters still remained significant after adjustment for other factors that cause prolonged cytopenia (ie, baseline cytopenia, CAR construct).^7^ Thus, perturbations of cytokines and inflammation are 1 possible explanation. Reduced levels of stromal cell-derived factor 1, a CXCR4 ligand that regulates hematopoietic stem cell migration and survival, neutrophil migration, and pro-pre-B cell development were implicated in delayed neutrophil recovery in 1 study.^2^ However, the analysis of multiple proinflammatory cytokines and chemokines as well as growth factors yielded no significant differences between the 8 patients with complete recovery at 1 month post-CART therapy besides macrophage-derived CCL22.^7^ Preexisting bone marrow damage seems to represent a risk factor for sustained myelosuppression after CART therapy as preexisting thrombocytopenia was associated with prolonged cytopenia and 2 of 8 patients showed signs of myelodysplasia in 1 study.^3^ Moreover, prior autologous or allogenic stem cell transplantation correlated with delayed recovery.^2,7^

Since patients with severe cytopenias after CART treatment are vitally endangered by bleeding and infection, ASCT seems a safe and feasible option to rescue bone marrow function in this setting.

## Disclosures

PG received travel support from Novartis and Gilead Sciences. JMH received travel support from Gilead Sciences. NK received research funding Gilead Sciences travel support from Gilead Sciences, Janssen, BMS/Celgene. BNB received travel support from Medac, Servier. CS received honoraria from BMS/Celgene, Gilead Sciences, Janssen, and Novartis. PB received honoraria from BMS/Celgene, Gilead Sciences, Janssen, Miltenyi Biotech, and Novartis. UH received honoraria from BMS/Celgene, Gilead Sciences, Janssen, Miltenyi Biotech, and Novartis.
